# Exploring the Potential of an Eye Tissue Donor Reporting App in Enhancing the Procurement of Corneal Donors: Mixed Methods Observational Study

**DOI:** 10.2196/50398

**Published:** 2024-05-15

**Authors:** Dominika Szkodny, Ewa Wróblewska-Czajka, Mikołaj Stryja, Filip Gara, Edward Wylęgała

**Affiliations:** 1 Chair and Clinical Department of Ophthalmology Faculty of Medical Sciences Zabrze Medical University of Silesia Katowice Poland; 2 Department of Ophthalmology District Railway Hospital Katowice Poland; 3 ArtNovation Gliwice Poland

**Keywords:** eye donor, corneal transplant, donor shortage, mHealth, mobile health, app, apps, applications, application, tissue procurement, organ, procurement, donor, donors, donation, transplant, transplantation, transplants, usability, experience, experiences, attitude, attitudes, opinion, perception, perceptions, perspective, perspectives, acceptance, interview, interviews, survey, surveys, questionnaire, questionnaires, reporting

## Abstract

**Background:**

The availability of donated eye tissue saves and enhances vision in transplant recipients; however, the current demand for tissue surpasses the available supply. Corneal donor shortages lead to increased wait times, delayed surgeries, prolonged visual impairment, and increased inconvenience to patients requiring eye tissue transplantation. A web-based application was previously developed to facilitate easy and intuitive submission of potential donor information.

**Objective:**

The primary objectives of this study were to assess health care professionals’ attitudes toward the potential application and evaluate its effectiveness based on user feedback and donor registrations through the application.

**Methods:**

Researchers used a mixed methods approach, commencing with a literature review to identify challenges associated with donor procurement. Stakeholder interviews were conducted to gauge health care professionals’ perspectives regarding the application. User feedback was collected through questionnaires, surveys, and interviews to assess the application’s usability and impact. An assessment of the reported potential donors and questionnaire responses were analyzed.

**Results:**

The final version of the application successfully reported 24 real cornea donors. Among 64 health care providers who used the application to communicate about potential donors, 32 of them submitted trial entries exclusively for testing purposes. The remaining 8 health care professionals reported potential donors; however, these individuals did not meet the donor qualification criteria. The majority of participants found the application user-friendly and expressed their readiness to use it in the future. Positive ratings were assigned to the layout, appearance, purpose, and specific features of the application. Respondents highlighted the automatic sending of notifications via SMS text messages and the integration of all necessary documents for donor qualification and tissue collection as the most valuable functions of the application.

**Conclusions:**

The study indicates that donor reporting applications offer promising solutions to enhance tissue donor procurement. This application streamlined the reporting process, reduced paperwork, facilitated communication, and collected valuable data for analysis.

## Introduction

The availability of donated tissues plays a crucial role in saving and enhancing the vision of numerous patients through transplantation. However, the demand for tissues consistently surpasses the available supply, leading to longer waiting lists and compromised patient outcomes in various countries, including Poland [1-3]. Challenges such as a lack of knowledge about donor reporting methods, eligibility criteria, insufficient financing, and limited distribution of eye banks further hinder the procurement of donated tissues [3,4]. To address this critical issue, concerted efforts and innovative approaches are essential to augment the pool of corneal donors. Various factors contribute to donor shortage, including a lack of awareness and education, cultural and religious beliefs, organizational and infrastructure challenges, and inadequate funding [2,3,5,6].

The shortage of corneal donors has profound consequences among individuals in need of corneal transplantation, leading to increased waiting times [7]. This, in turn, results in delayed surgeries, prolonged visual impairment, and heightened inconvenience to patients. In some instances, patients may be denied a transplant due to the unavailability of suitable donor corneas. Prolonged waiting times and the inability to perform timely corneal transplantation contribute to elevated health care costs. Individuals with visual impairments may require ongoing medical care, rehabilitation services, and assistive devices, placing a financial burden on both health care systems and individuals [8].

The availability of corneal donors is pivotal for advancing research and developing new technologies and treatments in the field of ophthalmology. Without an adequate supply of donor corneas, researchers and scientists face limitations in studying corneal diseases, developing innovative surgical techniques, and exploring new therapies [9]. Introducing multidirectional solutions is imperative to address and improve this challenging situation. One potential solution to alleviate corneal donor shortage is to automate the notification process for potential donors through a web-based application developed at our institution.

In this study, we explore the potential of using a donor reporting application as a means to enhance the tissue donation process.

## Methods

### Ethical Considerations

Ethical approval for this study was obtained from the institutional review board of the Medical University of Silesia (Katowice, Poland), and adherence to the tenets of the Declaration of Helsinki was ensured. Participation was voluntary, and informed consent was obtained from all participants before inclusion in the study. No personal data of the participants were collected, ensuring the anonymity and confidentiality of their responses. The questionnaire did not include any identifying information, and the article does not contain any individual participant information.

### Study Setting and Participant Recruitment

The study used a mixed methods approach, combining a questionnaire and an observational component. The target population was composed of doctors working in two hospitals in Katowice, Poland, where agreements were signed regarding the use of the donor reporting application. All doctors in these hospitals received invitations to participate in the study. Data collection occurred from December 2022 to June 2023, with approval obtained from both hospital directors. Doctors from the following departments ultimately took part in the study, because these departments have reported deaths and potential donors and used the application: cardiology, cardiac intensive care unit, neurology, anesthesiology and intensive care, and anesthesiology and intensive care with cardiology monitoring. The department heads from each aforementioned unit were provided with log-in credentials within the application and subsequently received a demonstration of its functionality. Paper-based surveys assessing the application’s performance were then distributed within the respective departments, additionally supplemented by an electronic version sent directly to their designated email addresses. The approximate number of doctors working in these departments is 120.

### Data Collection

A self-administered questionnaire evaluated the usability of the application (including the user interface, navigation, etc), design aesthetics and functionality, implementation feasibility within hospital workflows, and its potential impact on future donor reporting rates. The questionnaire did not rely on a specific technology acceptance framework; rather, it included a combination of closed-ended (Likert scale–rated, yes/no) and open-ended questions. The questionnaire contained 26 questions and is shown in Multimedia Appendix 1.

### Data Analysis

Data from the questionnaires were then collected and analyzed, with results presented as the number and percentage of people providing a particular response for qualitative variables, and the mean and SD for quantitative variables. The number of potential and final tissue donors was determined in the application from the tissue bank employee panel, to which notifications from the application were sent.

## Results

The final version of the application was version 3, and 24 real cornea donors were reported using the application. A total of 64 doctors sent messages through the application regarding potential donors (Table 1); however, 32 of them submitted only trial entries to test the application (with false data provided and a note indicating that the report was a test in the comments). The remaining 8 doctors reported potential donors; however, these individuals did not meet the donor qualification criteria (due to tumor markers or high procalcitonin). The estimated response rate was 50%.

Most participants found the application easy to use and expressed their willingness to use it in the future (Table 2). The layout, appearance, purpose, and specific features of the application received positive ratings (Table 3). The most valuable functions of the application indicated by the respondents was the automatic sending of notifications through SMS text message and integrating all necessary documents for donor qualification and tissue collection. The majority agreed that the application could encourage doctors to report tissue donors. Additional comments from users regarding the app included the following: “There is a need to work on the appearance of the application,” “The application is simple and intuitive,” “The automatic printing of the donor qualification form is a strong feature of the application,” “The most useful aspect is the automatic transmission of information to the tissue bank,” and “The application within the information system may get lost or not be prominently displayed.”

The interviewed users emphasized the importance of seamless integration with hospital systems. It has also been noted that data collection and reporting methods should be consistent to ensure reliable and comparable information across different regions or sites (Table 4).

**Table 1:** Participant demographics (N=64).

Characteristics		Participants, n (%)
**Age (years)**		
	18-30	35 (54.69)
	30-50	26 (40.62)
	>50	3 (4.69)
**Sex**		
	Male	24 (37.5)
	Female	40 (62.5)

**Table 2:** Users’ feedback regarding the application. The table shows the number of participants who answered “yes” to the question (N=64).

Feedback	Participants, n (%)
Would consider using the application in the future	57 (89.06)
Consider the application easy to use	56 (87.50)
Do not need technical support to navigate the application	55 (85.94)
Disagree with most doctors learning quickly	6 (9.38)
Do not need to learn new skills to use the application	59 (92.19)

**Table 3:** Application ratings on a 5-point scale.

Rating	Rating, mean
Layout	4.42
Appearance	4.10
Purpose	4.72
Generating donor qualification cards	4.86
Automatic notification to the eye tissue bank	4.94

**Table 4:** Application usage preferences. The table shows the number of participants who answered “yes” to the questions (N=64).

Preferences	Participants, n (%)
Would use the application on a workplace computer	60 (93.75)
The application can encourage doctors to report tissue donors	56 (87.50)
The application should be integrated with the hospital IT system and available in all hospitals	58 (90.63)

## Discussion

### Principal Findings

Several of our findings shed light on the performance and reception of the application. First, it is noteworthy that the application has proven successful, having reported 24 real cornea donors. This indicates a practical and tangible impact on the intended purpose of the application. Feedback from the participants is largely positive, with the majority expressing an inclination to use the application in the future. Ease of use is highlighted as a strong point, with a significant proportion of participants stating that they would not need technical support to navigate the application. The automatic notification feature and integration of necessary documents for donor qualification and tissue collection emerged as the most valuable functions of the application, as indicated by the respondents. This suggests that the application effectively streamlines and simplifies critical processes related to tissue donation. A vast majority of users expressed their willingness to use the application on their workplace computers, and there is a consensus that the application could encourage doctors to report tissue donors. However, the study also highlights areas for improvement. Interviewed users underscore the importance of seamless integration with hospital systems, suggesting that further efforts may be needed to enhance interoperability. Moreover, the application’s graphic design requires an upgrade, as it was the lowest-rated feature.

It is important to note that tissue donor procurement is faced with several challenges such as the lack of awareness and understanding among the general public regarding donation or underfinancing of eye banks [10-13].⁠ Many people are unaware of the tremendous impact tissue donation can have on others. This lack of awareness often leads to difficulties with talking to the family of a potential donor [14-18]. Public awareness campaigns serve as a cornerstone in increasing corneal donor procurement [19]. Additionally, the procurement process itself can be complex and confusing for doctors. These can delay the retrieval and transplantation of tissues, negatively impacting patient outcomes [20].⁠ This application optimized the recognition of tissue donors, as it specifies all eligibility criteria. It overcomes obstacles such as not knowing how to report a donor, which is an established factor contributing to inefficient recognition of tissue donors [10,21-23]. Positive feedback about the application is even more important because it has been proven that that users’ willingness to use it is influenced by their perceptions of both the application’s ease of use and its perceived usefulness [24]. Insights from participants can shape the future development of applications, particularly helping identify crucial elements for optimal design [25].

Donor reporting applications can collect and analyze data regarding donors’ demographics, registration trends, and geographical distribution. These insights can help organ procurement organizations and health care organizations identify areas with low donor registration rates, enabling targeted awareness campaigns to increase participation [26].

Although the application appears to be a promising tool for increasing the reporting of potential donors, it is essential to acknowledge certain limitations that may impact the interpretation of our findings. The study focused on doctors from 2 specific hospitals, potentially limiting the diversity of perspectives and experiences. Therefore, our findings may not be fully representative of the broader medical community. Furthermore, the use of a questionnaire for data collection introduces the possibility of response bias. Participants who chose to participate in the study may have unique characteristics or motivations that differ from those of nonparticipants. Finally, the data collection period, from December 2022 to June 2023, may not capture potential changes in user perceptions and experiences over an extended period.

### Conclusions

The study demonstrates a positive response to the donor reporting application, with promising implications for increasing cornea donations. The findings provide valuable insights into user preferences and highlight areas for refinement, ensuring the continued success and effectiveness of the application in the context of eye tissue donation. The developed program is a valuable tool that can significantly support the process of reporting potential tissue donors. It is worth noting that the effectiveness of the application has been confirmed by reports from actual cornea donors and positive user reviews.

## Appendix

Multimedia Appendix 1 The questionnaire used in the study.

Multimedia Appendix 2 Conversation with ChatGPT.

## Data Availability

The data sets generated during and analyzed during this study are available from the corresponding author on reasonable request.
