# Supplementary material for: Exploring the Potential of an Eye Tissue Donor Reporting App in Enhancing the Procurement of Corneal Donors: Mixed Methods Observational Study
Source: JMIR Form Res. 2024 May 15;8:e50398. doi: 10.2196/50398 (PMC11137423; doi:10.2196/50398)
Supplement: Multimedia Appendix 1 [file formative_v8i1e50398_app1.docx]

**1.Gender:**

a) Female b) Male

**2. Age:**

a) 18-30 years old b) 30-50 years old c) 50-70 years old d) >70 years old

**Questions 3-10 concern the usability of the system**

1. I think I would be happy to use this app. a) I agree b) I don't have an opinion c) I disagree
2. I think the app is easy to use. a) I agree b) I don't have an opinion c) I disagree
3. I think I would need technical support to use this app. a) I agree b) I don't have an opinion c) I disagree
4. I think the app is consistent and has no inconsistencies a) I agree b) I don't have an opinion c) I disagree
5. I imagine that most doctors would learn how to use this app very quickly. a) I agree b) I don't have an opinion c) I disagree
6. I feel very confident using the app. a) I agree b) I don't have an opinion c) I disagree
7. I would have to learn a lot before I can start using this app. a) I agree b) I don't have an opinion c) I disagree
8. Do you have any general comments on the usability of this app? Yes                          No Comment:

**Questions 11-16; each question uses a five-point answer format with formulations at the poles (e.g. question 11 "very unreadable" - 1, and "very readable" - 5)**

1. How readable is the layout of the app? 1              2                  3                  4              5
2. To what extent do you think the design meets the purpose of the app? 1              2                  3                  4              5
3. How user-friendly is the overall look and feel of the app? 1              2                  3                  4              5
4. How useful is the app's printable donor qualification card generation? 1              2                  3                  4              5
5. How useful is the automatic sending of a donor notification to the tissue bank? 1              2                  3                  4              5
6. To what extent do you feel the content of the app is sufficient to allow you to self-register a potential donor? 1              2                  3                  4              5
7. Do you have any general comments on the design and content of this app? Yes                          No Comment:

**Answers to questions 18-21 are "yes" or "no" with space for comments below.**

1. Is there anything you would add to this app to make it more useful in reporting a donor? Yes                          No Comment:
2. Is there anything in this app that you think is unnecessary or can be removed? Yes                          No Comment:
3. Is there anything you particularly liked about this app? Yes                          No Comment:
4. Is there anything you particularly disliked about this app? Yes                          No Comment:

**Questions 22-26 concern future use (answer "yes" or "no")**

1. Would you use this app if it were available on a computer at work? Yes                          No Comment:
2. Can this app encourage doctors to report tissue donors? Yes                          No Comment:
3. Should this app be integrated with the hospital information system (e.g. with AMMS)? Yes                          No Comment:
4. Should this app be available in every hospital? Yes                          No Comment:
5. Do you have any final comments on this app? Yes                          No Comment:
