# Supplementary material for: Exploring the Potential of an Eye Tissue Donor Reporting App in Enhancing the Procurement of Corneal Donors: Mixed Methods Observational Study
Source: JMIR Form Res. 2024 May 15;8:e50398. doi: 10.2196/50398 (PMC11137423; doi:10.2196/50398)
Supplement: Multimedia Appendix 2 [file formative_v8i1e50398_app2.pdf]

remaining 8 doctors reported potential donors; however, these individuals did not meet the donor qualification criteria (due to tumor markers or high procalcitonin).

ChatGPT

The third iteration marked the final version of the application, resulting in the reporting of 24 authentic cornea donors. Out of the 64 doctors who utilized the app to communicate about potential donors, 32 submitted trial entries exclusively for testing purposes. These trial entries involved the use of fictitious data, clearly indicated as a test in the comments. The remaining 8 doctors reported potential donors; however, these individuals did not meet the donor qualification criteria, primarily due to the presence of tumor markers or elevated procalcitonin levels.

Anonimowy

Most participants found the application easy to use and expressed a willingness to use it in the future. The layout, appearance, purpose, and specific features of the application received positive ratings. The most valuable functions on the app indicated by the respondents was the automatic sending of notification through the text message and integrating all necessary documents for donor qualification and tissue collection.

ChatGPT

The majority of participants found the application user-friendly and expressed a readiness to use it in the future. Positive ratings were given to the layout, appearance, purpose, and specific features of the application. Respondents highlighted the automatic sending of notifications via text messages and the integration of all necessary documents for donor qualification and tissue collection as the most valuable

Rozpocznij pracę z czatbotem ChatGPT

Zgłoś treść | Warunki użytkowania | Zasady ochrony prywatności |

przetłumacz: aplikacja działa w formie strony internetowej

ChatGPT

The application operates in the form of a website.

Anonimowy

dokonano również oceny zgłoszonych potencjlanych dawców oraz zgłoszeń testowych

ChatGPT

An assessment of the reported potential donors and test submissions was also conducted.

Anonimowy

prawdziwe zgłoszenia

ChatGPT

"Real submissions"

Anonimowy

zgłoesznia dawców przez aplikację

ChatGPT

"donor registrations through the application"

Anonimowy

The final version of the app was the third one and 24 real cornea donors were reported using the application. A total of 64 doctors sent messages through the app regarding potential [illegible]

Rozpocznij pracę z czatbotem ChatGPT

Zgłoś treść | Warunki użytkowania | Zasady ochrony prywatności |

In drawing conclusions, this study posits that donor reporting apps present highly promising solutions for the enhancement of tissue donor procurement processes. The app demonstrated notable efficiencies by streamlining the reporting procedure, minimizing paperwork, fostering improved communication, and concurrently amassing valuable data for subsequent analysis. This robust evaluation underscores the potential transformative impact of technology in optimizing critical aspects of the organ and tissue donation ecosystem.

Anonimowy
biorąc pod uwagę wyniki przeprowadzonych badań, wskazujących na znaczny niedobór zgłaszanych potnecjalnych dawców oraz na brak znajomości procedury ich zgłaszania stworzono aplikację umożliwjącą proste wykonanie takieg zgłoszenia

ChatGPT
W świetle wyników przeprowadzonych badań, które wyraźnie wskazywały na znaczny niedobór zgłaszanych potencjalnych dawców, oraz na brak znajomości procedury ich zgłaszania, stworzono aplikację umożliwiającą łatwe i intuicyjne zgłaszanie potencjalnych dawców.

Anonimowy
przetłumacz na angielski

ChatGPT
In light of the results of conducted research, which clearly indicated a significant shortage of reported potential donors and a lack of awareness regarding the procedure for their registration, an application has been developed to facilitate easy and intuitive submission of potential donor information.

Rozpocznij pracę z czatbotem ChatGPT

Zgłoś treść | Warunki użytkowania | Zasady ochrony prywatności |

?

enhance tissue donor procurement. The app streamlined the reporting process, reduced paperwork, facilitated communication, and collected valuable data for analysis.

ChatGPT

In this investigation, the focus was on assessing the potential of a donor reporting app to augment the tissue donation process, with a specific emphasis on corneal donors. A thorough literature review was undertaken to comprehensively comprehend the challenges and underlying reasons contributing to the shortages in donor availability.

The primary objectives of this study were multifaceted, involving the evaluation of healthcare professionals' attitudes toward the app and a meticulous assessment of its efficacy through detailed user feedback. To achieve these aims, a robust mixed-methods approach was employed, commencing with an in-depth literature review aimed at identifying challenges inherent in donor procurement. Subsequently, stakeholder interviews were conducted to gauge the nuanced perspectives of healthcare professionals regarding the app. The collection of user feedback was executed through a diverse array of methodologies, including questionnaires, surveys, and interviews, ensuring a comprehensive evaluation of the app's usability and overall impact.

The culmination of these efforts resulted in the successful reporting of 24 actual cornea donors through the final iteration of the app, with feedback gleaned from 64 users. Notably, participants consistently appraised the app for its user-friendly interface and expressed a noteworthy willingness to incorporate it into their future practices. Positive commendation was particularly directed toward the automatic notification feature and the seamless integration of necessary documents

Rozpocznij pracę z czatbotem ChatGPT

Zgłoś treść | Warunki użytkowania | Zasady ochrony prywatności |

?

it's important to note that the specific methodological details would determine the exact categorization.

Anonimowy

roboust this abstract: StrategiesHow to increase Tissue Donor Procurement:/ Exploring the Potential of Eye Tissue Donor Reporting App: observational study

ChatGPT

Enhancing Tissue Donor Procurement Strategies: An Observational Study Investigating the Efficacy of an Eye Tissue Donor Reporting App

Anonimowy

make this abstract mor robust: Background: This study aimed to explore the potential of a donor reporting app in enhancing the tissue donation process, specifically focusing on corneal donors. A comprehensive literature review was conducted to understand the challenges and reasons contributing to donor shortages.
Objectives: The primary objectives were to assess healthcare professionals' attitudes toward the potential app and evaluate its effectiveness through user feedback.
Methods: Researchers employed a mixed-methods approach, commencing with a literature review to identify challenges in donor procurement. Stakeholder interviews were conducted to gauge healthcare professionals' perspectives on the app. User feedback was collected through questionnaires, surveys, and interviews to assess the app's usability and impact.
Results: The final app version successfully reported 24 real cornea donors, garnering feedback from 64 app users. Participants generally found the app user-friendly and expressed a willingness to use it in the future. Positive ratings were given to the automatic notification feature and the integration of necessary documents.

Rozpocznij pracę z czatbotem ChatGPT

Zgłoś treść | Warunki użytkowania | Zasady ochrony prywatności |

4. **Conclusion:** The study concludes that donor reporting apps, exemplified by the specific app in question, offer promising solutions to enhance tissue donor procurement. The app is credited with streamlining the reporting process, reducing paperwork, facilitating communication, and collecting valuable data for analysis.

In summary, this study is an applied research effort that seeks to address a practical issue (tissue donor shortages) by implementing and evaluating a technological solution (donor reporting app) in a real-world context involving healthcare professionals and users.

Anonimowy

what kind of study is this - trial/ observational/ other

ChatGPT

The study described in the provided information is not explicitly labeled as a clinical trial or an observational study. However, it shares characteristics with an observational study, as it involves the collection and analysis of data without the explicit manipulation of variables by the researchers. The study employs a mixed-methods approach, including a literature review, stakeholder interviews, and user feedback collection, which aligns with observational research methods.

In an observational study, researchers observe and gather information about a phenomenon, in this case, the implementation and impact of a donor reporting app in the context of tissue donation. The study does not mention interventions, control groups, or randomization typically associated with clinical trials.

Rozpocznij pracę z czatbotem ChatGPT

Zgłoś treść | Warunki użytkowania | Zasady ochrony prywatności |

ChatGPT

Based on the provided information, this study falls under the category of applied research within the field of healthcare. Specifically, it focuses on the application and evaluation of a donor reporting app in the context of tissue donation, with a primary focus on corneal donors.

Here's a breakdown of the key elements:

1. **Objective:** The study aims to explore the potential of a donor reporting app in enhancing the tissue donation process, with a specific focus on corneal donors. The primary objectives are to assess healthcare professionals' attitudes toward the app and evaluate its effectiveness through user feedback.

2. **Methodology:** The researchers employed a mixed-methods approach, starting with a literature review to understand challenges in donor procurement. This was followed by stakeholder interviews to gather healthcare professionals' perspectives on the app. User feedback was collected through questionnaires, surveys, and interviews to assess the app's usability and impact.

3. **Results:** The study reports on the successful implementation of the app, which facilitated the reporting of 24 real cornea donors. Feedback from 64 app users is presented, indicating positive perceptions of the app's user-friendliness and a willingness to use it in the future. Positive ratings were given to specific features, such as automatic notifications and the integration of necessary documents.

Rozpocznij pracę z czatbotem ChatGPT

Zgłoś treść | Warunki użytkowania | Zasady ochrony prywatności |

Donor App Enhances Procurement

13 maja 2024

Anonimowy

what kind of study is this study: Abstract:
Background: This study aimed to explore the potential of a donor reporting app in enhancing the tissue donation process, specifically focusing on corneal donors. A comprehensive literature review was conducted to understand the challenges and reasons contributing to donor shortages.
Objectives: The primary objectives were to assess healthcare professionals' attitudes toward the potential app and evaluate its effectiveness through user feedback.
Methods: Researchers employed a mixed-methods approach, commencing with a literature review to identify challenges in donor procurement. Stakeholder interviews were conducted to gauge healthcare professionals' perspectives on the app. User feedback was collected through questionnaires, surveys, and interviews to assess the app's usability and impact.
Results: The final app version successfully reported 24 real cornea donors, garnering feedback from 64 app users. Participants generally found the app user-friendly and expressed a willingness to use it in the future. Positive ratings were given to the automatic notification feature and the integration of necessary documents.
Conclusions: The study concludes that donor reporting apps offer promising solutions to enhance tissue donor procurement. The app streamlined the reporting process, reduced

Rozpocznij pracę z czatbotem ChatGPT

Zgłoś treść | Warunki użytkowania | Zasady ochrony prywatności |
